# Clinical and human burden of respiratory syncytial virus in older adults: a narrative review with emphasis on Italian data

**DOI:** 10.1007/s40520-026-03424-1

**Published:** 2026-06-10

**Authors:** Nicola Veronese, Ligia Dominguez, Giovanni M. Giammanco, Pietro Giuseppe Innocenti, Ida Mancuso, Francesco Saverio Ragusa, Francesco Vitale, Mario Barbagallo

**Affiliations:** 1 Geriatric Unit, Department of Medicine, University of Palermo, Palermo, 90100 Italy; 2 Dipartimento di Promozione della Salute, Materno-Infantile, di Medicina Interna e Specialistica di Eccellenza G. D’Alessandro (PROMISE), Azienda Ospedaliera Universitaria Policlinico P. Giaccone-University of Palermo, Palermo, 90127 Italy; 3 Pfizer S.r.l, Roma, Italy

**Keywords:** Respiratory syncytial virus, Elderly, Hospitalization, Mortality, Sequelae

## Abstract

Respiratory syncytial virus (RSV) is a well-known driver of bronchiolitis and hospital admissions in infants. It is also a major threat for adults aged ≥ 60 years, causing acute respiratory infection with substantial morbidity and mortality. Only recently RSV surveillance programs have been implemented in Europe. In Italy, InfluNet, which has monitored influenza-like illness since 2000, began systematic RSV surveillance in the 2022–2023 season, now integrated within the national RespiVirNet framework. Interest in the burden of RSV infection in older patients has increased rapidly, particularly with new preventive tools. This narrative review summarizes recent evidence on the epidemiology and clinical features of RSV in older people, focusing on Italian data. The burden associated with hospitalization, functional and cognitive sequelae is emphasized, and comparisons with influenza are made. Evidence shows that RSV plays a significant role in winter hospital admissions among older adults, often leading to complications and lingering functional deficits beyond discharge. Those with preexisting conditions are especially at risk, and recent findings point to potential associations with cognitive deterioration. Numerous studies reveal that the severity of RSV-related hospitalisations matches that seen with influenza. Although more robust and harmonized data are needed, current evidence supports implementing targeted prevention strategies to lessen the impact of RSV in older adults. An integrated approach—combining rapid and sensitive diagnostics, routine surveillance, and access to preventive measures such as immunisation, particularly for frail individuals—holds promise for reducing the burden of RSV infection and alleviating pressures on national health services.

## Background and objectives

Respiratory syncytial virus (RSV) is a ubiquitous respiratory pathogen in the family Pneumoviridae (genus Orthopneumovirus). Two antigenically and genetically distinct subgroups, RSV-A and RSV-B, circulate each year and can be distinguished by molecular features [1]. Like other seasonal respiratory viruses, RSV shows predictable annual waves. In temperate countries, including Italy, transmission intensifies in winter, typically peaking between December and January [2]. While RSV is a well-known driver of bronchiolitis and hospital admission in infants, it is also a major threat for older adults and adults with underlying comorbidities, and is an important cause of acute respiratory infection resulting in substantial morbidity and mortality [3]. Recognition of this burden has grown over the last two decades. In a landmark prospective study of community cohorts and high‑risk adults with chronic cardiac or pulmonary disease, RSV accounted for a clinically meaningful fraction of winter respiratory illness, with an impact comparable to non‑pandemic influenza A in populations with high influenza vaccine uptake [4]. In older adults, upper airway infection may progress to lower respiratory tract disease, precipitate pneumonia, and trigger exacerbations of underlying heart and lung conditions [5]. More recently, RSV has been reported to cause morbidity and in-hospital mortality that may meet or exceed that of influenza among hospitalized older adults [6]. These considerations also explain why RSV is increasingly viewed as a core pathogen in geriatric respiratory medicine rather than a niche diagnosis.

This narrative review summarizes current evidence on the epidemiology and clinical features of RSV in older people, with particular attention to Italy. The Italian focus is justified by several contextual factors: Italy has one of the oldest populations in Europe; its temperate climate shapes RSV seasonal dynamics; and influenza-like illness monitoring has a long-standing national infrastructure (InfluNet) that can support respiratory virus surveillance. Where relevant, we compare RSV with influenza, another major winter virus in geriatrics, to highlight shared clinical challenges and important differences that matter for diagnosis, management, and prevention in later life. In this framework, we also consider how recent changes in European and national surveillance—such as the inclusion of RSV into broader respiratory virus platforms—may support estimates, and how these estimates can be translated into decisions on testing, hospitalization pathways, and prevention for adults at highest risk.

### Data sources and search strategy

We searched PubMed, Embase, and the Cochrane Library, and screened relevant geriatric and infectious-disease journals, from inception to 01 December 2025. Search strings combined free-text terms and controlled vocabulary (e.g., MeSH) and included “respiratory syncytial virus”, “RSV”, “influenza”, “elderly”, “older adults”, “long-term hospitalization”, “functional impairment”, “cognitive decline”, “dementia”, and “frailty”, adapting syntax to each database.

Eligible records were English-language studies and reviews that addressed RSV in older adults or provided age‑stratified data relevant to older adults. We prioritized evidence describing clinical outcomes, hospital burden, and post‑hospital deficits, and included comparative studies of RSV versus influenza, as well as work specifically describing the Italian setting. We acknowledge that a detailed flow of study selection (including the number of records identified, screened, and included) was not systematically recorded during the search process, given that this was a narrative review. Articles were included that were deemed of relevance.

## Epidemiology of RSV in older adults

RSV is estimated to cause more than 30 million lower respiratory tract infections worldwide each year and around 3 million hospitalizations [7]. The incidence of medically attended RSV rises steadily with age among adults and is highest among people aged ≥ 70 years [3]. Quantifying the incidence in adults has long been difficult because clinical presentation overlaps with other winter viruses and RSV testing is inconsistently performed. A systematic review and meta‑analysis in high‑income countries estimated that in 2019 adults aged ≥ 60 years experienced over 5 million acute RSV infections, approximately 466,000 hospitalizations, and 33,000 in‑hospital deaths; in Europe alone, the estimates were > 3 million infections, ~ 274,000 hospitalizations, and ~ 19,500 deaths [8]. Case fatality increases with age and comorbidity and has been reported as high as 7% in adults aged ≥ 60 years in high‑income settings [6]. Importantly, these totals capture only in-hospital deaths and may not fully reflect excess mortality occurring after discharge or in long‑term care facilities.

In Italy, several recent studies provide insight into the epidemiology of RSV in older adults (Table 1). In a multicentre prospective investigation during the 2018–2019 pre‑pandemic season in northern and central regions, RSV positivity among outpatients of all ages was 6.4%, but with a clear clinical burden in older patients [9]. A large retrospective analysis from Rome (8,836 nasopharyngeal samples, 2016–2023) found an overall RSV positivity of 17.2%, with the second highest prevalence in older adults (age ≥ 65 years) [10]. In primary care, in the two-season RESPIRA‑50 study (adults ≥ 50 years), of 1,431 patients included 5.2% tested positive for RSV, with slightly higher rates among patients meeting acute respiratory infection definitions than those meeting influenza‑like illness definitions [11, 12]. In a review focusing on the epidemiological and clinical impact of RSV in older adults in Europe, 8 of the 25 studies included were carried out in Italy; the prevalence of RSV in Italy was 8.4–9.9% in adults aged 65–80 years, rising to 10.2–13.8% in those aged > 80 years [13]. Overall, the available data from Italy suggest a prevalence that may be somewhat higher than that reported across other European countries.

**Table 1:** Summary of Italian epidemiological data on RSV infection prevalence, and correlated hospitalization and mortality rates

Author, year	Type of study	Reference period	Centers/investigators involved	Sample	Outcomes	Limitations
**Prevalence rates**						
Pierangeli, 2023 [9]	Prospective, multicenter study	Dec 2018– Apr 2019	8 GLIViRe* centers	1,300 RSV+ cases	• RSV-positivity 6.4% in all ages, most samples being of adult patients • RSV-A > RSV-B in patients ≥ 65 years • Substantial disease burden was demonstrated in older patients	• Limited to one season
Liotti, 2025 [10]	Retrospective analysis	Sep 2016– Aug 2023	IRCCS** Policlinico Universitario A. Gemelli, Rome	8,836 nasopharyngeal samples	• RSV-positivity 17.2% (all ages) • Age-stratified analysis revealed RSV predominance in infants, followed by older adults (≥ 65 years)	• Retrospective, single-center design • External factors such as vaccination rates not accounted for
Domnich, 2025 [11]	Prospective community-based study comparing ARI° to ILI°° (RESPIRA-50 study; first season results)	2023–2024 RSV season	24 GPs^	517 subjects ≥ 50 years with ARI (250) or ILI (267)	• 7.0% tested positive for RSV • RSV prevalence was higher in the ARI group (8.0%) than in the ILI group (6.0%)	• Small number of RSV-positive samples led to wide 95% CIs • Limited sample size during the first season
Domnich, 2025 [12]	Prospective community-based study comparing ARI to ILI (RESPIRA-50 study; second season results)	2023–2025 RSV seasons	38 GPs	1,431 patients ≥ 50 years with ARI (741) or ILI (690)	• 5.2% tested positive for RSV • 5.8% in the ARI group and 4.6% in the ILI group	• Unequal cluster sizes and comparatively low circulation of RSV reduced statistical power • RSV-ARI case definition derived from a comparatively low number of RSV cases was not validated externally
D’Ambrosio, 2025 [13]	Comprehensive review of epidemiology of RSV among elderly and high-risk adult populations in Europe	2015–2025		- 25 studies included - 8/25 in Italy (32%)	• Italian prevalence: o 8.4-9.9% (65–80 years) o 10.2–13.8% (> 80 years)	• Studies included in review had heterogeneity in terms of designs, settings, and outcomes • Lack of standardized case definitions across different surveillance systems
Bracaloni, 2024 [14]	Multiregional study on nasopharyngeal swabs	Nov 2022– Apr 2023	Enrolment by GPs Analyses by University centers of Genova, Pisa, and Bari	152 patients with ARI > 65 years	• 21.7% tested positive for RSV • 6.1% were serotype A • 93.9% were serotype B	• Small sample size and challenges in GP participation • Late start missed early seasonal data
Hak, 2025 [37]	Prospective cohort study	2022–2024 RSV seasons	GPs (Italy and The Netherlands)	703 adults ≥ 65 years presenting with ARI tested for RSV and influenza	• RSV-positivity 13.2% • Mean age 76 years • 63% had ≥ 1 comorbidity • Annual RSV-ARI incidence rate was 10.3 episodes per 1000 person-years	• Selection bias may have influenced findings • Differences in RSV-positivity rates between Italy and Netherlands could reflect differences in diagnostic methods
**Hospitalization**,** ICU**^**+**^ **admission**,** and mortality rates**						
Méroc, 2024 [35]	Analysis of a database predisposed by CREA Sanità^§^	2015–2019	Epidemiology and statistical centers		• 10,931,573 cardiorespiratory hospitalizations (adults) • 1,390,267 deaths (adults) • Older adults aged ≥ 65 years accounted for 74% and 94% of all cardiorespiratory hospitalizations and deaths, respectively	• Monthly counts, were used, limiting the number of time points included • The RSV activity proxy used hospitalization data from children aged < 2 years, which may not reflect the exact temporality of circulation of the virus in the adult population
Bracaloni, 2024 [14]	Multiregional study on nasopharyngeal swabs	Nov 2022– Apr 2023	Enrolment by GPs Analyses by University centers of Genova, Pisa, and Bari	152 patients with ARI > 65 years	• 3% required hospitalization	• Small sample size and challenges in GP participation • Late start missed early seasonal data
D’Ambrosio, 2025 [13]	Comprehensive review of epidemiology of RSV among elderly and high-risk adult populations in Europe	2015–2025		25 studies included 8/25 in Italy (32%)	• In Italy, 33.3% of the sample reported ≥ 1 annual hospitalization • The mortality rate was 7–9% in patients aged ≥ 65 years, with an estimate of 7.1% for individuals aged ≥ 70 years	• Studies included in review had heterogeneity in terms of designs, settings, and outcomes • Lack of standardized case definitions across different surveillance systems
Hak, 2025 [37]	Prospective cohort study	2022–2024 RSV seasons	GPs (Italy and The Netherlands)	703 adults ≥ 65 years presenting with ARI tested for RSV and influenza	• Emergency department referral in 5% (4/88) of RSV patients • Hospitalization in 2% (2/88) of RSV patients	• Selection bias may have influenced findings • Differences in RSV-positivity rates between Italy and Netherlands could reflect differences in diagnostic methods
Domnich, 2025 [11]	Prospective community-based study comparing ARI to ILI (RESPIRA-50 study; second season results)	2023–2025 RSV seasons	38 GPs	1,431 patients ≥ 50 years with ARI (741) or ILI (690)	• Among the 75 RSV-positive patients o case-hospitalization rate was 2.7% o case-fatality rate was 1.3%	• Small number of RSV-positive samples led to wide 95% CIs • Limited sample size during the first season

* Gruppo di Lavoro sulle Infezioni Virali Respiratorie (Working Group on Respiratory Virus Infections)

** Istituto di Ricovero e Cura a Carattere Scientifico (Scientific Institute for Hospitalization and Care)

° Acute Respiratory Infection: °° Influenza-Like Illness

^ General Practitioners

^§^ The CREA Sanità database is predisposed by extracting monthly data from the Italian hospitalization collection data of the Ministry of Health and the Italian National Institute of Statistics (ISTAT) data (mortality)

^+^ Intensive Care Unit

Patterns of subtype circulation in older adults also appear age‑dependent. Across Italian and European reports, the relative proportions of RSV-A and RSV-B vary by season, but RSV-B frequently predominates in the oldest age groups. The relative contribution of RSV-A and RSV-B in older adults also appears variable across seasons and settings. In an Italian multicenter study, Pierangeli et al. reported an overall prevalence of 18.7% for RSV-A and 23.9% for RSV-B, with slightly higher prevalence in individuals > 80 years compared with those aged 65–80 years (10.2% vs. 8.4% for RSV-A; 13.8% vs. 10.1% for RSV-B) [9]. However, in a separate Italian primary-care pilot study conducted in adults aged ≥ 65 years, Bracaloni et al. observed a marked predominance of RSV-B among RSV-positive ARI cases (94% RSV-B vs. 6% RSV-A), underscoring how subtype predominance can shift substantially between studies and contexts [14].

Despite increasing recognition, RSV in older adults remains underdiagnosed. Surveys and reviews suggest that many clinicians acknowledge RSV as an issue, but report limited familiarity with its epidemiology and prevention, and patient awareness is generally lower than for influenza or COVID‑19 [15, 16]. As adult RSV vaccines become available, accurately defining disease burden in people aged > 60 years becomes essential for planning implementation and evaluating impact. This requires surveillance that adequately samples older adults. Historically, most RSV surveillance programs in Europe and globally have focused on children, leaving adults under‑represented. RSV was only recently incorporated into the European Surveillance System (TESSy) [17]. In Italy, InfluNet has monitored influenza-like illness since 2000, whereas systematic RSV surveillance began only in the 2022–2023 season [18]. RSV monitoring is now integrated within the national RespiVirNet framework that is coordinated by the Istituto Superiore di Sanità (ISS) [19, 20]. However, key limitations of RespiVirNet include reliance on upper‑respiratory swab sampling, the seasonal and time‑limited nature of data collection (which may miss early/late circulation and reduces comparability across seasons), limited age resolution within older adults (with all individuals aged ≥ 65 years grouped together, without further stratification), potential heterogeneity across regions and sites in sampling intensity and operational implementation, given the decentralized regional organization of recruitment, sampling logistics, and laboratory workflows. In any case, the currently-available data from other analyses are insufficient to fully characterize clinical impact in older adults as highlighted in a multiregion pilot study [14]. Consequently, influenza-like illness surveillance alone may substantially underestimate the burden of RSV in geriatric populations. In the European RESCEU cohort, the WHO ILI case definition captured only 11% of PCR-confirmed RSV infections in adults aged ≥ 60 years, underestimating the occurrence of RSV by up to 9-fold, largely because RSV-associated respiratory infections in this population frequently present without fever [21]. Diagnostic testing practices represent an additional source of under-ascertainment in adults: a systematic review and meta-analysis estimated that reliance on nasopharyngeal swab RT-PCR alone misses 28–52% of RSV infections depending on the type of specimen [22], a finding confirmed by a multi-specimen prospective study reporting that RSV detection more than doubled when multiple specimen types were used [23]. These data indicate that the true burden of RSV in older adults is considerably higher than current surveillance estimates suggest. Harmonized case definitions and testing strategies, aligned with influenza and COVID-19 programs, would likely improve comparability across regions and over time.

## Clinical characteristics of RSV in older adults

Compared with pediatric RSV, the clinical picture in older adults is less well described, particularly in community and outpatient settings. Symptom onset usually occurs 2–8 days after exposure. Typical manifestations include cough, rhinorrhea, sneezing, fever, wheezing, sore throat, headache, congestion, and fatigue [24] . In practice, RSV, influenza A/B, and SARS‑CoV‑2 can present with overlapping symptom clusters—including fever, cough, and dyspnea—making syndromic diagnosis unreliable [25]. For pragmatic counselling, the National Foundation for Infectious Diseases has provided a comparative symptom profile for flu, RSV, COVID‑19, and the common cold (Table 2) [26].

**Table 2:** Clinical differences between Flu, RSV, COVID-19, and the Common Cold [26]. Common symptoms may include cough, headaches, sneezing, runny nose, and congestion. Different symptoms may include the following

Symptom	COLD	FLU	RSV	SARS-CoV-2
Aches	XX	XXX	X	XX
Difficulty breathing	X	X	XX	XXX
Fatigue	XX	XXX	X	XXX
Fever	X	XXX	XX	XX
Loss of taste or smell	X	X	X	XXX
Sore throat	XXX	XX	X	XXX
Wheezing	X	X	XXX	X

X: rarely; XX: sometimes; XXX: often

In older adults, RSV-associated disease often affects the lower respiratory tract and may lead to respiratory failure, which is seen in 8–13% of cases [27]. Beyond respiratory symptoms, clinicians have reported that acute RSV in older people may be accompanied by disorientation and marked shortness of breath [24]. Across all studies, prevalent comorbidities in RSV‑infected older adults include cardiovascular disease (about 53–59%), diabetes (around 30–31%), chronic obstructive pulmonary disease (roughly 17–29%; COPD), and immunosuppression (up to 38%) [28–30]. In clinical practice, distinguishing viral from bacterial disease is challenging, and RSV-associated radiographic pneumonia is not uncommon in hospitalized seniors.

Italian observational data similarly identify chronic cardiopulmonary and metabolic disease as being common in RSV cases, with COPD (≈ 38%), diabetes (≈ 22%), and heart failure (≈ 15%) among the most frequent comorbidities [31]. RSV infection often precipitates exacerbations of pre‑existing airway disease: worsening asthma has been reported in up to 64.9% of cases and COPD exacerbations in as many as 83.0% [32]. Importantly, the relationship appears bidirectional – patients with asthma or COPD also have a higher likelihood of severe RSV illness [32].

## Hospitalization due to RSV in older adults

RSV is often cited as the third most common viral cause of hospitalization in older adults, with the greatest impact in those aged ≥ 65 years [2]. In high‑income countries, early estimates suggested ~ 157,000 RSV-related hospitalizations annually among adults, corresponding to roughly 157 admissions per 100,000 population in people aged ≥ 65 years. More recent modelling that adjusts for under‑ascertainment suggests the burden is substantially higher: nearly 800,000 RSV‑associated hospitalizations occurred in adults aged ≥ 65 years in 2019 across high‑income settings [33]. These figures underscore that severe RSV is a major driver of winter respiratory admissions in later life.

European estimates are consistent with a substantial hospitalization burden. A systematic analysis by the Respiratory Virus Global Epidemiology Network reported that, in the 5 European countries with sufficient direct data, adjusted annual RSV hospitalization rates in adults aged ≥ 60 years ranged from 193 to 414 per 100,000 person‑years. For countries lacking robust direct estimates, an ensemble model predicted rates of 223 to 317 per 100,000 person‑years [34]. In-hospital case fatality also varied by country, from 6.7% in Spain to 10.1% in Switzerland [34].

Italian estimates of burden have been refined by modelling approaches that move beyond RSV‑specific ICD coding. Using CREA Sanità hospitalization records and ISTAT mortality data, Méroc et al. estimated that between 2015 and 2019 the annual incidence of RSV‑attributable cardiorespiratory hospitalizations in adults aged ≥ 75 years ranged from 1,064 to 1,527 per 100,000 person‑years—substantially higher than estimates based solely on RSV diagnostic codes [35]. In a multiregional primary-care study (Liguria, Apulia, Tuscany), hospitalization occurred in ~ 3% of ARI cases in adults aged > 65 years [14]. Among hospitalized Italian patients with RSV (mean age 73.7 years), the average length of stay was 17 days, intensive care unit (ICU) admission occurred in 5.8%, and in-hospital mortality was 9.1% [31]. The economic burden is also considerable: mean direct healthcare costs in RSV-hospitalized adults aged ≥ 60 years approached €12,000, largely driven by ward and ICU stay, and were ~ 15% higher than costs for patients hospitalized for any reason [36]. In prospective primary-care cohorts of adults aged ≥ 60 years tested for RSV and influenza, RSV positivity ranged from 13.2% in a larger cohort to similar levels in smaller studies; follow-up showed frequent repeat visits (38%), emergency referral (≈ 5%), and hospitalization in about 2–3% of RSV-positive cases, with low but non‑zero mortality [12, 37]. These findings suggest that even when hospitalization rates from primary care appear low, the subset who deteriorate can consume substantial resources because of long stays and high comorbidity burden.

Residence in long‑term care facilities substantially increases the risk and severity of RSV infection. In US data, older adults living in skilled nursing facilities or assisted living facilities had a higher incidence of hospitalization for RSV infection than community dwellers (mean annual estimates of ~ 440 and ~ 740 per 100,000, respectively, versus ~ 117 per 100,000 in community settings) [38]. A systematic review of studies in nursing and care homes (2000–2023) reported an annual incidence of RSV-positive lower respiratory tract infection around 3,000 per 100,000 residents, with RSV‑ARI hospitalization rates approximately 600–1,100 per 100,000 person‑years [39]. The higher mortality observed in these settings likely reflects advanced age, frailty, and multimorbidity among residents.

## Long-term outcomes of RSV infection in older adults

Older adults with RSV—particularly those who are frail or have multiple comorbidities—are at high risk of severe disease, prolonged hospitalization, and death [6]. RSV can aggravate COPD, asthma, and congestive heart failure, contributing to complications and mortality [30, 40]. Emerging evidence also highlights diabetes as a frequent underlying condition associated with worse in‑hospital outcomes in adults aged ≥ 60 years [41]. Beyond pulmonary effects, RSV has been linked to increased cardiovascular risk, and associations with acute events and longer‑term cardiovascular morbidity and mortality have been described [42].

Italian follow‑up data underline the potential for serious post‑acute outcomes. In a retrospective cohort of adults aged ≥ 50 years hospitalized for RSV infection between 2010 and 2021 and followed for up to 12 months, approximately 8% experienced stroke and 3.3% acute myocardial infarction. During follow‑up, nearly half showed worsening of pre‑existing comorbidities, while rehospitalization (44%) and mortality (30%) were high [31].

A broader question is whether respiratory infections contribute to neurocognitive decline. Although mechanistic hypotheses exist, epidemiologic evidence remains limited. In a review of RSV in nursing and care homes, cognitive impairment and dementia were among the most commonly reported chronic conditions, suggesting substantial vulnerability in this population [39]. In a population-based study evaluating seropositivity to RSV (and other pathogens) in older adults (mean age 67.6 years), RSV seropositivity showed a modest but statistically significant association with mild cognitive impairment and dementia [43]. Hospitalization for respiratory infection is also a recognized trigger for functional decline in older adults, often persisting beyond the acute episode [44, 45]. Specific longitudinal RSV data are still scarce, but the available studies indicate risk increases with age and frailty [4, 42, 46]. In a cohort of 302 older adults, RSV hospitalization led to acute deterioration on activities-of-daily-living scales; one third had persistent ADL impairment at 6 months and 14% required higher levels of care at discharge [47]. Post‑discharge surveillance in the CDC‑funded IVY Network (26 hospitals, 20 states) also found functional impairment in about 22% of adults after RSV hospitalization, with surprisingly small differences by age group [48]. A systematic review and meta‑analysis of 21 studies (1990–2019) reported persistent functional decline as the most frequent sequela within one year (33.5%, 95% CI 27.6–39.9), along with substantial healthcare utilization such as rehospitalization and transfer to skilled nursing facilities [49]. For clinicians, these data emphasize that recovery trajectories after RSV are often prolonged and that discharge planning should anticipate rehabilitation needs, caregiver support, and the possibility of institutionalization in the frailest individuals.

## Comparative data with influenza

Contrasting data has been published comparing outcomes with RSV to influenza. Hospitalized older patients with RSV often develop pneumonia, require ventilatory support, and face risks that extend beyond the acute phase, including persistent physical or cognitive impairment and elevated long‑term mortality. In estimates for the Global Burden of Disease Study (2010), influenza was associated with roughly 500,000 deaths annually and RSV with about 250,000; among older adults, mortality risk after hospitalization for RSV was similar to that for influenza [50]. A systematic review of observational studies reported that RSV-attributable hospitalization incidence in older adults is broadly similar to influenza [51]. In the U.S. HARTI study, influenza was diagnosed more often, but RSV patients tended to be older (mean 67.3 years) and to have more comorbidities, with greater healthcare resource use [52]. Other work has found comparable severity between RSV and influenza admissions [53], while some reports suggest RSV hospitalizations may be more severe despite similar complication profiles [25, 54]. In a meta-analysis up to June 2022 including 762,084 older adults, the risk of hospitalization and mortality did not differ significantly between RSV and influenza when assessed as cumulative incidence or rates [51]. In another study comparing RSV-positive patients to influenza-positive patients, those infected with RSV were more likely to be older, more frequently prescribed antibiotics, and more often admitted to hospital [55]. Mortality was also substantially higher in RSV-positive patients (15% vs. 6%).

Italian emergency‑department data support the view that RSV can be at least as severe as influenza in frail patients. In a retrospective cohort from L. Sacco University Hospital (Milan) including adults presenting with flu‑like symptoms or acute respiratory failure, RSV cases had the highest rates of acute respiratory failure (62.7%) and severe disease (70.5%). Mortality in RSV patients was similar to influenza A (6.6% vs. 5.9%) [56]. Compared with influenza A, RSV patients were older, had higher Charlson comorbidity indices, and more chronic heart failure [56].

Taken together, current evidence indicates that RSV in older adults may frequently be at least as severe as influenza, and may even be more severe in selected settings. The burden is concentrated among people with cardiovascular disease and chronic respiratory disorders, in whom RSV is associated with substantial morbidity, prolonged hospitalization, and non‑trivial mortality.

Nonetheless, it is acknowledged that being a narrative review, the present analysis has some limitations as it is potentially subjective and may be affected by selection bias compared to a systematic review. Another limitation is that the available data is also subject to potential bias related to the time‑limited nature of data collection, potential heterogeneity across regions and sites in sampling.

## Conclusions

Until recently, RSV was largely viewed as a pediatric pathogen, and adult disease has been relatively neglected. Limited outpatient testing, nonspecific symptomatology, and surveillance systems designed around influenza-like illness have contributed to under‑recognition. Methodological heterogeneity across studies has further obscured the true burden in adults. Nevertheless, interest has increased rapidly, particularly with the arrival of new preventive tools. The evidence reviewed here shows that each winter RSV contributes substantially to hospital admissions among adults aged ≥ 60 years, with frequent complications and with downstream functional consequences that can persist after discharge. Older adults with chronic respiratory or cardiovascular disease are particularly vulnerable, and emerging data suggest possible links with cognitive decline. Multiple studies—internationally and in Italy—indicate that RSV hospitalizations can be comparable in severity to those caused by influenza. Although more robust and harmonized data are needed, the current literature supports targeted prevention strategies aimed at reducing RSV impact in older populations.

Improving the response to RSV in later life will require an integrated approach. First, clinicians need easy access to rapid and sensitive diagnostics in emergency departments, wards, and primary care so that RSV can be identified alongside influenza and SARS‑CoV‑2 and managed appropriately, including antimicrobial stewardship, supportive care planning, and infection‑control measures. Second, surveillance should routinely sample older adults—both community dwellers and long‑term care residents—and feed timely data back to clinicians and public health authorities to guide resource allocation during winter peaks. Third, analytic approaches that estimate attributable burden (e.g., cardiorespiratory admissions) are needed, because reliance on RSV-specific ICD codes undercounts disease. Finally, equitable access to preventive interventions, including immunization where indicated, should be paired with post‑implementation monitoring of uptake, effectiveness, and safety, particularly in frail and multimorbid patients. Coordinated action across these domains can mitigate clinical impact, protect high‑risk groups, and strengthen health-system resilience to seasonal respiratory virus pressure.

## Data Availability

No original data were used for this work.
